# SIMULATING WELL-BEING AND LITERACY INTERVENTIONS TO REDUCE ELDER SCAM SUSCEPTIBILITY

**DOI:** 10.1093/geroni/igac059.738

**Published:** 2022-12-20

**Authors:** Marguerite DeLiema, Aparajita Sur

**Affiliations:** University of Minnesota, Twin Cities, Minneapolis, Minnesota, United States; University of Minnesota, Minneapolis, Minnesota, United States

## Abstract

Finanical fraud targeting older adults is on the rise, with annual losses in the billions of dollars. There is little longitudinal research on the causal relationships between known risk factors and scam susceptibility, including poor psychological well-being and poor health and financial literacy. Interventions designed to enhance well-being and/or literacy may reduce scam susceptibly among older adults. In this study, we use repeated measures from the Rush Memory and Aging Project to simulate how different trajectories in well-being and literacy might impact scam susceptibility among older adults alive over a seven year period. We simulated the effects of interventions of varying degrees -- 10%, 50%, and 100% increase in well-being/literacy from baseline scores. Simulations were performed for all participants as well as by education and income subgroups. Simulation models show that intervening on well-being causes a greater reduction in average scam susceptibility over time compared to intervening on total literacy. Even a 10% increase in baseline well-being significantly reduces scam susceptibility over time, regardless of participants’ baseline income or educational attainment. Both interventions caused slightly greater reductions in susceptibility for those who are not college educated and those with an annual household income of less than $30,000. This study suggests that interventions that target self-efficacy and sense of purpose may help reduce older adults’ scam susceptibility even more than interventions that improve health and financial literacy, but that both are promising targets for intervention.

